# Diffuse thyroid enlargement and its regression in methotrexate-associated lymphoproliferative disorder: an ultrasonographic observation

**DOI:** 10.1530/EDM-25-0062

**Published:** 2025-12-23

**Authors:** Shingo Murasawa, Fumiya Isozaki, Shinobu Takayasu, Kazunori Kageyama, Yukihiro Fujita, Makoto Daimon

**Affiliations:** Department of Endocrinology and Metabolism, Hirosaki University Graduate School of Medicine, Hirosaki, Japan

**Keywords:** thyroid, malignant lymphoma, ultrasonography, methotrexate, hypothyroidism

## Abstract

**Summary:**

A 70-year-old woman with Hashimoto thyroiditis, treated with levothyroxine, was diagnosed with rheumatoid arthritis 1 year prior and subsequently began methotrexate (MTX) therapy. She presented with a 2-week history of progressive, painless anterior neck swelling. Ultrasonography (US) revealed diffuse thyroid enlargement with heterogeneously decreased echogenicity, and laboratory tests showed an elevated thyroid stimulating hormone (TSH) level of 63.6 mIU/L. A core needle biopsy confirmed the presence of mucosa-associated lymphoid tissue (MALT) lymphoma. Given her MTX therapy, MTX-associated lymphoproliferative disorder (MTX-LPD) was strongly suspected. Accordingly, MTX was discontinued, and her levothyroxine dosage was increased. Serial US examinations subsequently demonstrated a gradual reduction in thyroid size, and this progressive regression ultimately confirmed the diagnosis of MTX-LPD. Although MTX-LPD is a recognized complication of MTX therapy in rheumatoid arthritis, its primary occurrence in the thyroid is rare. In previous reports, primary thyroid lymphoma was either diagnosed in patients with a preexisting diagnosis of Hashimoto thyroiditis or concurrently with it. However, in our case, she already had Hashimoto thyroiditis, and the onset of MTX-LPD was accompanied by a worsening of hypothyroidism. Most patients with MTX-LPD achieve remission following MTX withdrawal. This rare case documents the natural improvement of diffuse ultrasonographic findings in thyroid MTX-LPD, highlighting the usefulness of serial ultrasonographic monitoring in patient follow-up.

**Learning points:**

- Patients receiving MTX therapy may develop MTX-LPD involving the thyroid gland.
- Thyroid MTX-LPD may present with concomitant worsening of hypothyroidism.
- Ultrasonographic findings in thyroid MTX-LPD can occasionally include diffuse thyroid enlargement.
- MALT lymphoma may occur as the histological subtype in primary thyroid MTX-LPD.
- MTX-LPD is expected to regress following discontinuation of MTX.
- Serial ultrasonographic monitoring of thyroid size can serve as an effective guide for assessing MTX-LPD regression.

## Background

Methotrexate (MTX) is widely used as a primary treatment for rheumatoid arthritis (RA) (1, 2). However, it can induce MTX-associated lymphoproliferative disorder (MTX-LPD) (3). In primary thyroid lymphoma, Hashimoto thyroiditis is often already established or diagnosed concurrently with the lymphoma diagnosis; however, to our knowledge, there have been no reports of cases in which preexisting hypothyroidism worsened in conjunction with the diagnosis of MTX-LPD, as observed in our case. The histological types of primary thyroid malignant lymphoma are MALT lymphoma and diffuse large B-cell lymphoma (DLBCL), each accounting for approximately half of the cases (4). Although the number of cases is small, both histological types have been reported in thyroid MTX-LPD (5, 6, 7, 8, 9). Furthermore, previous reports indicate that thyroid MTX-LPD is predominantly a nodular lesion on ultrasonography (US), with no reports of diffuse enlargement (8, 9).

The initial management of MTX-LPD is discontinuation of MTX, with chemotherapy reserved for cases that do not achieve remission following MTX withdrawal (10). Although the thyroid gland is an uncommon site for MTX-LPD, in this report we describe a case diagnosed during MTX therapy in which diffuse thyroid enlargement improved after MTX discontinuation, as documented by serial US.

## Case presentation

A 70-year-old woman with Hashimoto thyroiditis, maintained on levothyroxine 25 μg/day, was diagnosed with RA 1 year earlier and subsequently began MTX therapy (the dose was 8 mg/week for the first 6 months and then reduced to 6 mg/week due to liver damage). She had been receiving tocilizumab 400 mg/4 weeks for the past 6 months. She presented with a 2-week history of progressive, painless anterior neck swelling. No B symptoms – namely, fever, night sweats, or unexplained weight loss – were observed in the patient. There was no history of iodine excess: the patient had no recent iodinated contrast exposure, no use of amiodarone or iodine-containing supplements (including kelp/seaweed products), no povidone–iodine use, and no occupational exposure; the dietary history was unremarkable for excessive iodine intake. Furthermore, no preceding symptoms or signs of thyrotoxicosis were elicited (e.g., palpitations, heat intolerance, tremor, or weight loss). Given the rapid thyroid enlargement observed during MTX therapy, we suspected the occurrence of MTX-LPD.

## Investigation

**Figure 1:**
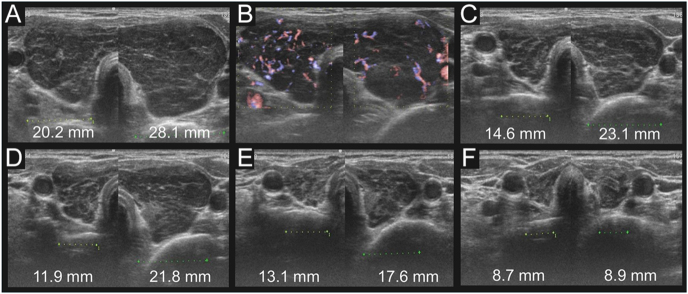
Ultrasonographic changes in the patient with thyroid MTX-LPD after MTX withdrawal. At the first visit, the thyroid showed diffuse enlargement and decreased echogenicity. (A) Color Doppler ultrasonography demonstrated diffuse, moderately increased vascularity. (B) The transverse diameter of each lobe (the numbers in the figure represent measurements in mm) was measured at the first visit and at 2-, 4-, 8-, and 12-months. (A, C, D, E, F) It showed significant shrinkage over time.

**Figure 2:**
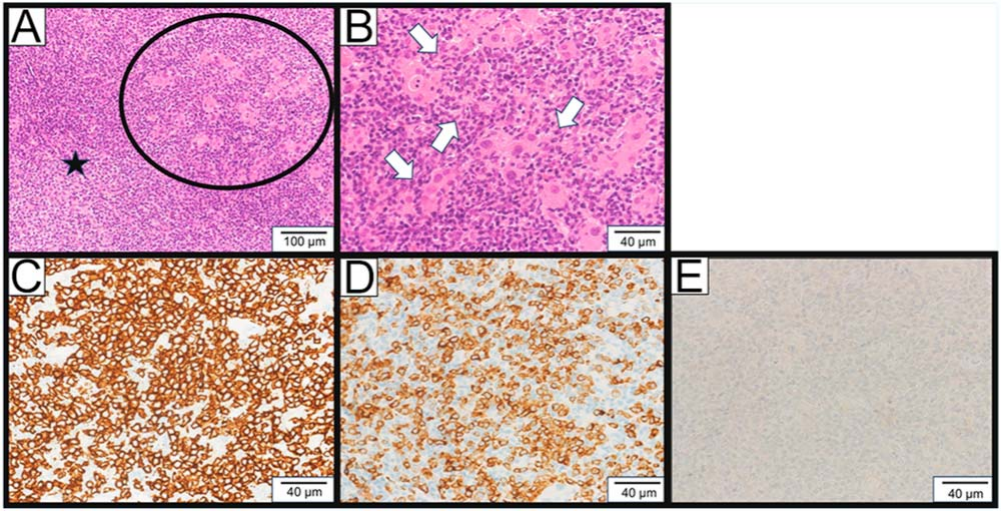
Histopathology of the thyroid gland. (A and B) H&E stain. Diffuse lymphocytic infiltration within the interstitium (star), with frequent destruction of the follicular epithelium (black circle) (A) and the presence of lymphoepithelial lesions (white arrows) (B), in which small lymphocytes infiltrate and disrupt the follicular epithelium. (C and D) Positive immunohistochemistry of IHC stain of CD20 (C) and CD79a (D). (E) Negative immunohistochemistry of IHC stain of CD3, CD5, and CD10.

US demonstrated diffuse thyroid enlargement with heterogeneously decreased echogenicity (Fig. 1A). Color Doppler US demonstrated diffuse, moderately increased vascularity of the thyroid gland (Fig. 1B), and several enlarged cervical lymph nodes were noted. No nodular lesions were observed in the thyroid. Whole-body computed tomography (CT) revealed no additional mass lesions. [18F]fluorodeoxyglucose positron emission tomography (FDG PET-CT) was not performed. Laboratory tests were consistent with hypothyroidism, showing an elevated TSH level of 63.6 mIU/L (reference range (RR): 0.61–4.23 mIU/L) and decreased free T3 (2.05 pg/mL; RR: 2.48–4.14 pg/mL) and free T4 (0.64 ng/dL; RR: 0.76–1.65 ng/dL). The data from 2 months earlier were as follows (TSH: 4.92 mIU/L, FT4: 1.12 ng/dL). Both anti-thyroid peroxidase antibody (223 IU/mL; RR: 0–15 IU/mL) and anti-thyroglobulin antibody (196 IU/mL; RR: 0–27 IU/mL) were positive, whereas the soluble interleukin-2 receptor (sIL-2R) was within the RR (157–474 U/mL) at 375 U/mL. The absolute lymphocyte count (ALC) in peripheral blood was 1,400/μL. Serological testing revealed positivity for Epstein–Barr virus (EBV) VCA IgM and IgG. EBV-DNA quantitative testing was not performed. A core needle biopsy of the left thyroid lobe was performed, and histopathological examination revealed mucosa-associated lymphoid tissue (MALT) lymphoma (Fig. 2). The biopsy showed diffuse lymphocytic infiltration within the interstitium, with frequent destruction of the follicular epithelium and the presence of lymphoepithelial lesions. Immunohistochemistry demonstrated diffuse positivity for CD20, CD79a, and BCL2, while CD10, BCL6, CD5, CD3, and CD138 were negative. These findings strongly suggested that the patient had thyroid MTX-LPD. Flow-cytometric assessment of κ/λ light-chain restriction and immunoglobulin heavy-chain (IGH) gene rearrangement testing to confirm B-cell monoclonality were not performed in this case.

## Treatment

After discussion with our rheumatology colleagues, we elected to discontinue MTX and manage the patient with clinical observation. Tocilizumab was also discontinued, and azulfidine 1,000 mg/day was started. Following MTX withdrawal, the patient was monitored regularly, and her levothyroxine dosage was gradually increased, ultimately reaching 125 μg/day.

## Outcome and follow-up

Over 1 year, serial US demonstrated a gradual reduction in thyroid size (Fig. 1C, D, E, F). After discontinuing MTX, the observed regression of thyroid enlargement confirmed a final diagnosis of MTX-LPD. No worsening of RA was observed after discontinuing MTX.

TSH, free T3, and free T4 returned to the reference range. The ALC exceeded 1,000/μL at 6 months post-onset. The sIL-2R was transiently elevated to 738 U/mL but subsequently normalized. EBV IgM seroconverted to negative, while EBV IgG remained positive.

## Discussion

Since the patient presented with hypothyroidism at onset, the thyroid enlargement could have been attributable to hypothyroidism alone. However, given her MTX therapy, we suspected MTX-LPD. A core needle biopsy was subsequently performed, and histopathological evaluation confirmed a diagnosis of MALT lymphoma. According to Watanabe *et al.*, Hashimoto thyroiditis was diagnosed in 154 (90%) of 171 cases of primary thyroid malignant lymphoma, and in 80 cases (47%), Hashimoto thyroiditis had been diagnosed before the identification of lymphoma (4). In 74 cases, Hashimoto thyroiditis was diagnosed concurrently with lymphoma. To the best of our knowledge, no cases have been reported in which worsening preexisting hypothyroidism occurred concurrently with a diagnosis of thyroid lymphoma. We hypothesize that the hypothyroidism resulted from the replacement of normal thyroid tissue by MTX-LPD. The improvement in thyroid enlargement is thought to be due to discontinuation of MTX-LPD.

Kurita *et al.* reported that in the 219 patients with MTX-LPD, primary thyroid involvement was observed in 5 cases (2.3%) (3). Watanabe *et al.* reported that 80 (47%) of 171 cases of primary thyroid lymphoma were MALT lymphoma (4). Mariette *et al.* reported that, among 25 cases of MTX-LPD, only one case was of primary thyroid origin; the histological type was diffuse large B-cell lymphoma (DLBCL) (5). In addition, a few cases of primary thyroid MTX-LPD have been documented (5, 6, 7, 8, 9); among these, two were DLBCL, and one had indeterminate histology. Suzuki *et al.* reported that 7 of 11 cases of primary thyroid MTX-associated lymphoproliferative disorder were MALT lymphoma (9). Our case, which demonstrated MALT lymphoma histology, further supports the findings by Suzuki *et al.* that MALT lymphoma may be a common histological subtype in primary thyroid MTX-LPD.

The initial treatment for MTX-LPD is discontinuation of MTX (10). The clinical course following MTX withdrawal may vary, including regression, relapse/regrowth, or no change; hence, careful follow-up is warranted. In this case, both EBV IgG and IgM were positive, suggesting a relatively recent EBV infection or reactivation. Previous studies have shown that patients with RA exhibit increased EBV viral loads and higher titers of EBV-specific antibodies compared to healthy individuals (11). Moreover, MTX has been implicated in the development of LPD by promoting the reactivation and proliferation of EBV-infected B cells via immunosuppression (12). EBV-positive LPD is reportedly more likely to regress following MTX discontinuation than EBV-negative LPD (13). Although neither EBV-DNA quantification nor EBER-ISH was performed in this case, the presence of both EBV IgG and IgM suggests possible EBV involvement. The observed regression of the lesion after discontinuing MTX is consistent with previous reports of EBV-positive MTX-LPD. Tokuhira *et al.* reported that the ALC is a useful predictor of remission after MTX discontinuation in MTX-LPD (14). In their study, an ALC of ≥1,000/μL at both onset and 6 months later was identified as a significant predictor of treatment-free survival. In the present case, the ALC exceeded 1,000/μL at both onset and 6 months post-onset, suggesting a high likelihood of achieving remission following MTX withdrawal.

The American College of Rheumatology 2021 guideline issues a conditional recommendation favoring rituximab over other disease-modifying antirheumatic drug (DMARDs) for patients with a previous LPD (1). In Japan, the use of rituximab for RA treatment is not covered by health insurance. However, the 2024 guidelines from the Japan College of Rheumatology state that clinical trials regarding the use of rituximab for patients with RA are currently underway (2). Nakano *et al.* reported on the safety and efficacy of using DMARDs after discontinuing MTX in patients with MTX-LPD (15). Both MTX and tocilizumab were discontinued; nevertheless, no clinical worsening of RA was observed, which may be attributable – at least in part – to the addition of azulfidine.

As a classification of ultrasonographic findings for primary thyroid lymphoma, Stein SA *et al.* defined three categories: nodular type, diffuse type, and mixed type (16). Tokumaru *et al.* described a case of primary thyroid MTX-LPD with nodular lesions that resolved on serial US (8). Zhang *et al.* also reported 11 cases of primary thyroid MALT lymphoma that were followed by serial US, demonstrating either spontaneous remission or a slowly progressive course (17). In this study, the US findings were nodular type in five cases, diffuse type in three, and mixed type in three. Suzuki *et al.* also reported that all 11 cases of thyroid MTX-induced lymphoproliferative disorder were nodular type (9). In our case, the thyroid did not show a nodular pattern; instead, it exhibited diffuse enlargement with uniformly hypoechoic parenchyma. Unlike previously reported cases, the hypoechoic lesion persisted after MTX withdrawal. Following MTX cessation, the diffuse enlargement improved, and both lobes decreased in size similarly. This course suggests that MTX-LPD may have involved the gland diffusely; however, the reduction in gland size could also be attributable to levothyroxine replacement in the context of elevated TSH. It cannot be excluded that a preexisting hypoechoic lesion enlarged under TSH stimulation and later regressed, or that a hypoechoic nodular lesion developed on the background of a diffusely hypoechoic thyroid. Considering the possibility of residual hypoechoic lesions, we cannot conclusively assert remission of MTX-LPD.

For initial staging and response assessment in Hodgkin and most non-Hodgkin lymphomas, FDG PET-CT has been incorporated into the Lugano classification as a standard modality (18).

Thyroid US is a simple, minimally invasive examination for patients, and it can be easily repeated. Although our case improved with MTX withdrawal alone, worsening thyroid enlargement may consider chemotherapy or alternative interventions. It is possible that microscopic lesions may not be detected based on imaging findings alone, and it is not possible to determine with certainty whether MTX-LPD lesions have truly disappeared. However, if serial US can detect recurrence of thyroid enlargement at the earliest stage, it becomes possible to suspect a relapse of MTX-LPD. Thus, ultrasonographic monitoring provides a practical and reliable method for follow-up of primary thyroid MTX-LPD after MTX discontinuation.

## Declaration of interest

The authors declare that there is no conflict of interest that could be perceived as prejudicing the impartiality of the research reported.

## Funding

This research did not receive any specific grant from any funding agency in the public, commercial, or not-for-profit sector.

## Patient consent

Written informed consent for publication of their clinical details and clinical images was obtained from the patient.

## Author contribution statement

SM drafted the original manuscript. ST, KK, YF, and MD critically reviewed and revised the manuscript for intellectual content. FI contributed to the collection of clinical data. All authors approved the final version of the manuscript for publication.
